# Metabolomics Reveals Differences in Muscle Metabolite Profiles Between Daweishan Mini Chickens and Tegel Broilers at Marketable Age

**DOI:** 10.3390/biology15201804

**Published:** 2026-10-09

**Authors:** Wei Huang, Jinshan Ran, Shangwen Wu, Jing Li, Tiao Ning, Yanli Du, Xiujuan Yang

**Affiliations:** 1 College of Agronomy and Life Sciences, Kunming University, Kunming 650214, China; huangwei_kmu@163.com (W.H.); lijing@kmu.edu.cn (J.L.);; 2 Faculty of Animal Science and Technology, Yunnan Agricultural University, Kunming 650201, China

**Keywords:** Daweishan mini chicken, Tegel broiler, untargeted metabolomics, differential metabolites, meat quality

## Abstract

Metabolic differences are an important factor influencing the quality of chicken meat. To investigate the differences in muscle flavor precursors between Daweishan mini chickens and Tegel broilers, integrated metabolomics analyses were used to compare the muscle metabolite profiles of the two breeds at marketable age (150-day-old Daweishan mini chickens and 45-day-old Tegel broilers). The differential metabolites (DMs) were significantly enriched in aminoacyl-tRNA biosynthesis; glycine, serine and threonine metabolism; glutathione metabolism; alanine, aspartate and glutamate metabolism; arginine biosynthesis; purine metabolism; pantothenate and CoA biosynthesis; cysteine and methionine metabolism; glycerophospholipid metabolism; and D-glutamine and D-glutamate metabolism, which contributed to the quality differences between the two breeds. Moreover, 3-methylhistidine, L-carnosine, L-anserine, DL-lactate, choline, L-phenylalanine, and L-ascorbic acid were present at higher relative abundances in the muscle of Daweishan mini chickens at marketable age. This study provides basic data on the mechanisms underlying the meat quality of local chickens and a reference for the development and utilization of local chicken breeds and the selective breeding of high-quality broilers.

## Simple Summary

The flavor and nutritional quality of chicken meat are largely determined by its chemical composition, which varies between different breeds and production systems. In this study, we compared the muscle metabolite profiles—the small molecules that contribute to taste, aroma, and nutritional value—between Daweishan mini chickens, a local Chinese indigenous breed raised for 150 days, and Tegel broilers, a commercial fast-growing breed raised for only 45 days. Using advanced metabolomics technology, we identified significant differences in many flavor-related metabolites between the two breeds. Key taste-active compounds, including L-carnosine, L-anserine, and certain amino acids, were found at much higher levels in the meat of Daweishan mini chickens. Our findings offer useful information for breeding programs aimed at improving broiler meat traits and for promoting the value of local chicken breeds.

## 1. Introduction

Chicken is highly favored by consumers due to its high nutritional value and affordable price. With the continuous improvement in living standards, consumers have increasingly higher demands for chicken flavor, taste, and health benefits, driving the broiler industry towards higher quality and differentiation. Many local chicken breeds have gradually gained consumer preference due to their tender, delicious meat and superior flavor. Chicken quality is comprehensively influenced by multiple factors [1,2,3]. Flavor is a complex process resulting from the combined action of various metabolites and chemical reactions, yet existing research seldom focuses on changes in metabolites, particularly the variations and regulatory mechanisms of small-molecule metabolites related to flavor, such as amino acids, fatty acids, and nucleotides [4]. Some scholars have conducted certain studies on the meat quality of local chicken breeds, especially regarding their amino acid composition and meat quality traits. However, most of these studies are limited to localized indicators and lack systematic metabolomic analysis.

Metabolomics focuses on the study of endogenous small molecules within organisms, which play crucial roles in maintaining normal functions and growth and development by participating in processes such as signal transduction, metabolism, energy supply, regulation of enzyme activity, and defense. This technology systematically analyzes changes in metabolites under external stimuli or different cellular states and examines the correlation between metabolite abundance and phenotypic characteristics, thereby revealing the relationship between external environments and the abundance of different differential metabolites [5,6]. Metabolomics is closely associated with phenotypes; it not only captures subtle changes at the genetic and protein levels but also reflects actual changes occurring within organisms more directly compared with genomics and proteomics. For instance, Ge et al. [5] used metabolomic analysis to reveal the formation mechanism of meat quality and flavor in Beijing-You chickens; Wang et al. [7] employed metabolomics to identify metabolites in the muscles of Lueyang black-boned chickens reared in cage and non-cage systems, discovering that the non-cage system favors the accumulation of flavor substances and nutrients in chicken meat. Huang et al. [8] used untargeted metabolomics to identify 20 metabolites that regulate the specific deposition of inosine monophosphate (IMP). Marco et al. [9] found that the digestible ratio of arginine (Arg) to lysine (Lys) affects energy and protein metabolism in broilers by regulating metabolites. Shi et al. [10] employed LC-MS technology and discovered that glycerophospholipid metabolism plays a key role in muscle flavor and development but exhibits different antioxidant patterns in different skeletal muscles.

The Daweishan mini chicken is a characteristic local breed native to Yunnan Province, China. It is a dual-purpose (meat and egg) local breed with ornamental and game value, characterized by slow growth and small body size. It possesses strong ornamental value and produces tender, flavorful meat; it also has well-developed breast and leg muscles, a plump body, and strong stress resistance, making it an excellent local chicken breed in China. The Tegel broiler is a commercial composite broiler line bred by the Tegel Poultry Development Company (Ingham Enterprises Pty Limited, North Ryde) in Australia, characterized by a fast growth rate, a high survival rate, strong adaptability, and high feed efficiency. In this study, Daweishan mini chickens and Tegel broilers were used as experimental animals. LC-MS technology was employed to analyze the differences in breast muscle metabolites between the two chicken breeds at market age at slaughter in order to screen differential metabolites associated with meat quality. The findings will provide a theoretical basis at the metabolic level for the exploitation of local chicken germplasm resources, breeding of high-quality broilers and improvement of meat quality.

## 2. Materials and Methods

### 2.1. Animals and Sample Collection

The experiment was carried out at the experimental chicken farm of Kunming University. All animal experiments were approved by the Animal Care and Use Committee of Kunming University, China (approval No. Kmu2025005). A total of 30 Daweishan mini chickens (DM group) and 30 Tegel broilers (DG group), with half males and half females in each group, were reared under identical dietary and feeding-management conditions. All birds were raised in individual cages. Each bird represented one experimental unit. All chickens were provided with ad libitum access to feed and drinking water. The indoor housing temperature was maintained at 22–26 °C and the relative humidity was 55–65%. The lighting regimen was 16 h light:8 h dark, with light intensity of 5–10 lx at the bird’s head level. The basal diet was formulated according to the nutritional requirements for local chicken breeds and commercial broilers, with corn-soybean meal as the main raw material. During the first 30 days, the dietary metabolizable energy was 12.60 MJ/kg, and crude protein was 20.55%. After 30 days of age, the metabolizable energy was 12.80 MJ/kg, and dietary crude protein was 18.35%. At marketable age (Daweishan mini chickens, 150 days; the mean body weight was 1122.20 ± 148.17 g; Tegel broilers, 45 days; the mean body weight was 2150.03 ± 191.01 g), 4 male and 4 female chickens within the range of the group mean body weight from each breed were selected for slaughter. Prior to slaughter, all experimental chickens were fasted for 12 h with free access to water. Birds were bled via carotid artery exsanguination, followed by defeathering, and breast-muscle tissues were rapidly dissected. Breast muscle samples (100 mg) were collected from each chicken, placed into RNase-free tubes, immediately frozen in liquid nitrogen, and stored at −80 °C.

### 2.2. Metabolomic Analysis of Muscle Tissue

#### 2.2.1. Sample Pretreatment

Samples stored at −80 °C were retrieved, and 60 mg of each sample was weighed. All samples were processed in a completely random order during sample pretreatment to eliminate processing-order bias. Then, 200 μL of water was added for MP homogenization, followed by vortexing for 60 s. Next, 800 μL of a methanol–acetonitrile solution (1:1, *v*/*v*) was added, and the mixture was vortexed for another 60 s. The samples were subjected to low-temperature ultrasonication twice for 30 min each and then incubated at −20 °C for 1 h to precipitate proteins. After centrifugation at 14,000× *g* and 4 °C for 20 min. Quality-control (QC) samples were prepared by pooling equal volumes of supernatant from all breast-muscle test samples and processed identically to experimental samples. The obtained supernatants (including QC samples) were freeze-dried. The processed samples were stored at −80 °C.

#### 2.2.2. Chromatography–Mass Spectrometry Analysis

The samples were separated using an Agilent 1290 Infinity ultra-high-performance liquid chromatography (UHPLC) system (Santa Clara, CA, USA) equipped with a hydrophilic interaction liquid chromatography (HILIC) column at a column temperature of 25 °C and a flow rate of 0.3 mL/min. The mobile phase consisted of (A) water containing 25 mM ammonium acetate and 25 mM ammonia and (B) acetonitrile. The gradient elution program was as follows: 0–1 min, 95% B; 1–14 min, B decreased linearly from 95% to 65%; 14–16 min, B decreased linearly from 65% to 40%; 16–18 min, B maintained at 40%; 18–18.1 min, B increased linearly from 40% to 95%; 18.1–23 min, B maintained at 95%. Prior to injection, all samples were randomized for LC-MS acquisition. During the entire analysis, samples were kept in the autosampler at 4 °C and were analyzed in a random order to avoid fluctuations in instrument detection signals. Quality control (QC) samples were inserted into the sample sequence to monitor and evaluate system stability and the reliability of the experimental data.

Detection was performed using electrospray ionization (ESI) in both positive and negative ion modes. After UHPLC separation, samples were analyzed using a Triple TOF 5600 mass spectrometer (AB SCIEX) (Framingham, MA, USA). The ESI source conditions were as follows: Ion Source Gas1 (Gas1), 60; Ion Source Gas2 (Gas2), 60; Curtain gas (CUR), 30; source temperature, 600 °C; and IonSpray Voltage Floating (ISVF), ±5500 V in both positive and negative modes. The TOF MS scan range was *m*/*z* 60–1000, the product ion scan range was *m*/*z* 25–1000, the TOF MS accumulation time was 0.20 s/spectrum, and the product ion accumulation time was 0.05 s/spectrum. Secondary mass spectrometry was performed using information-dependent acquisition (IDA) in high sensitivity mode with declustering potential (DP) of ±60 V and collision energy of 35 ± 15 eV in both ion modes. The IDA settings were as follows: exclude isotopes within 4 Da, and monitor six candidate ions per cycle.

#### 2.2.3. Metabolomics Quality Control and Data Pre-Processing

Feature reproducibility was evaluated by calculating the relative standard deviation (RSD) of metabolite peaks across QC samples; metabolic features with RSD > 30% in QC samples were excluded from downstream analysis. For missing-value handling, features with missing values exceeding 50% within any experimental group were discarded. The remaining missing values were imputed using the k-nearest-neighbor (KNN) algorithm. Total-ion-intensity normalization was carried out for all metabolite intensities to correct differences originating from sample loading. Feature filtering criteria were set as follows: metabolic features were retained only if they were detected in at least 50% of samples in at least one group and exhibited QC-sample RSD ≤ 30%. The filtered dataset was used for subsequent univariate and multivariate statistical analyses.

#### 2.2.4. Data Processing

Raw data were converted to mzXML format using ProteoWizard (version 3.0) and then processed with the XCMS program for peak alignment, retention time correction, and peak area extraction. Metabolite identification was performed using accurate mass matching (<25 ppm) and secondary spectral matching against the laboratory in-house database.

After Pareto-scaling, the data were subjected to orthogonal partial least squares discriminant analysis (OPLS-DA) for multivariate statistical analysis. Univariate statistical analysis included Student’s *t*-test and fold change analysis. OPLS-DA modeling was implemented with the R package ropls, and 200-iteration permutation tests were carried out to assess model reliability. The actual R^2^ and Q^2^ values of each OPLS-DA model are provided in the results. An OPLS-DA model was regarded as acceptable when the Q^2^ intercept from the permutation test was less than 0. Model VIP values were computed via multiple cross-validation. Metabolites satisfying both multivariate-statistical VIP > 1 and univariate-statistical *p* < 0.05 were defined as significantly differential metabolites. Metabolites with VIP > 1 and 0.05 < *p* < 0.1 were regarded as marginally differential metabolites. The hypergeometric distribution test was applied for KEGG pathway enrichment analysis of differential metabolites. R software (version 4.3.1) was used to generate heatmaps and correlation plots.

## 3. Results

**Figure 1:**
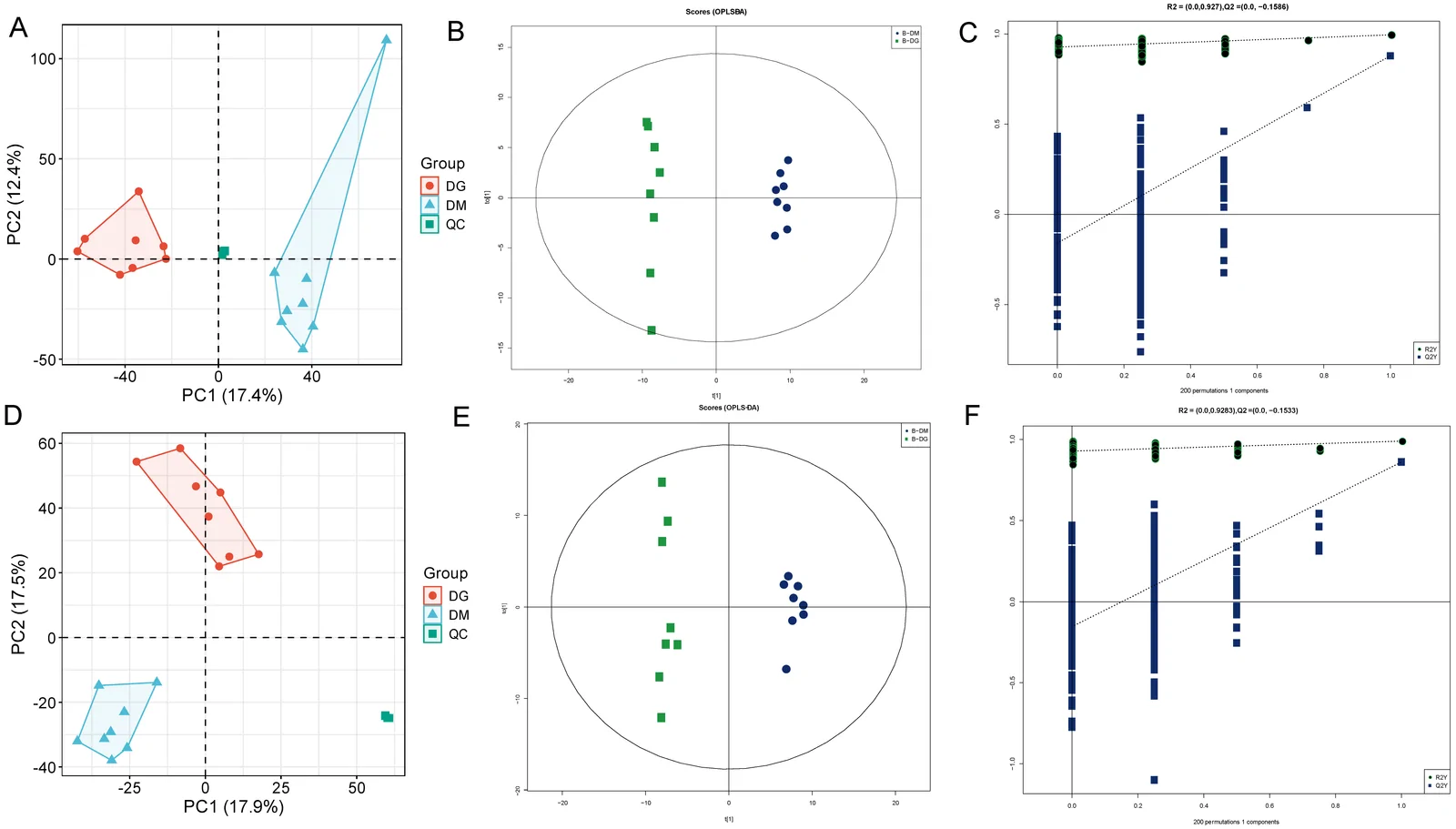
Metabolome quality control analysis. (**A**) PCA in positive ion mode; (**B**) OPLS-DA score plot in positive ion mode; (**C**) permutation test in positive ion mode; (**D**) PCA in negative ion mode; (**E**) OPLS-DA score plot in negative ion mode; (**F**) permutation test in negative ion mode. Note: In the OPLS-DA score plots, the horizontal and vertical axes represent the interpretation of the predictive component(1) and orthogonal component to(1), each point represents a sample, and colors indicate groups. R2 (explained variance) and Q2 (predictive ability) values were used to evaluate model classification performance. In the permutation test plots, the model is considered reliable and valid if all blue Q2 points are lower than the rightmost original Q2 point.

To evaluate the separation of samples from the two breeds and to screen key metabolites, OPLS-DA was conducted. Under positive-ion (POS) mode, the cumulative R^2^X, R^2^Y and Q^2^ of the OPLS-DA model were 0.301, 0.995 and 0.879, respectively. Under negative-ion (NEG) mode, the cumulative R^2^X, R^2^Y and Q^2^ were 0.316, 0.990 and 0.861, respectively (Table 1). The DM and DG samples, as well as with QC, were clearly separated (Figure 1A,D), and the OPLS-DA models showed good fit and predictive ability (Figure 1B,E). Permutation testing indicated that the models were valid and reliable (Figure 1C,F).

**Figure 2:**
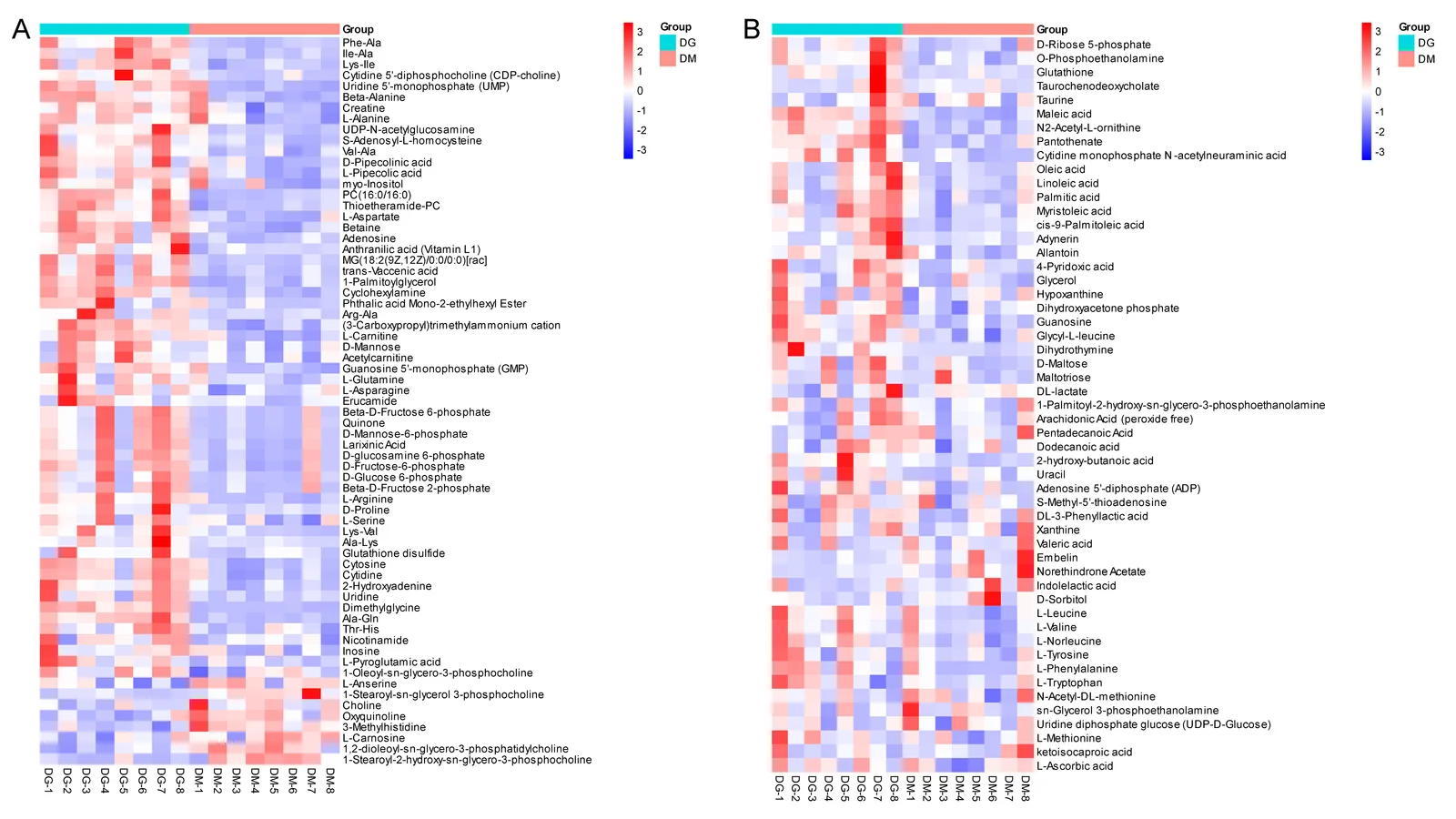
Heatmaps of DMs in (**A**) positive and (**B**) negative ion mode.

Differential metabolites (DMs) were visualized by hierarchical clustering analysis to show the relationships between samples and the differences in the expression patterns of DMs across samples (Figure 2). In positive ion mode, 9 DMs were upregulated and 59 DMs were downregulated in Daweishan mini chickens compared with Tegel broilers (Figure 2A; Table S1). In negative ion mode, 8 DMs were upregulated and 53 DMs were downregulated (Figure 2B; Table S2).

**Figure 3:**
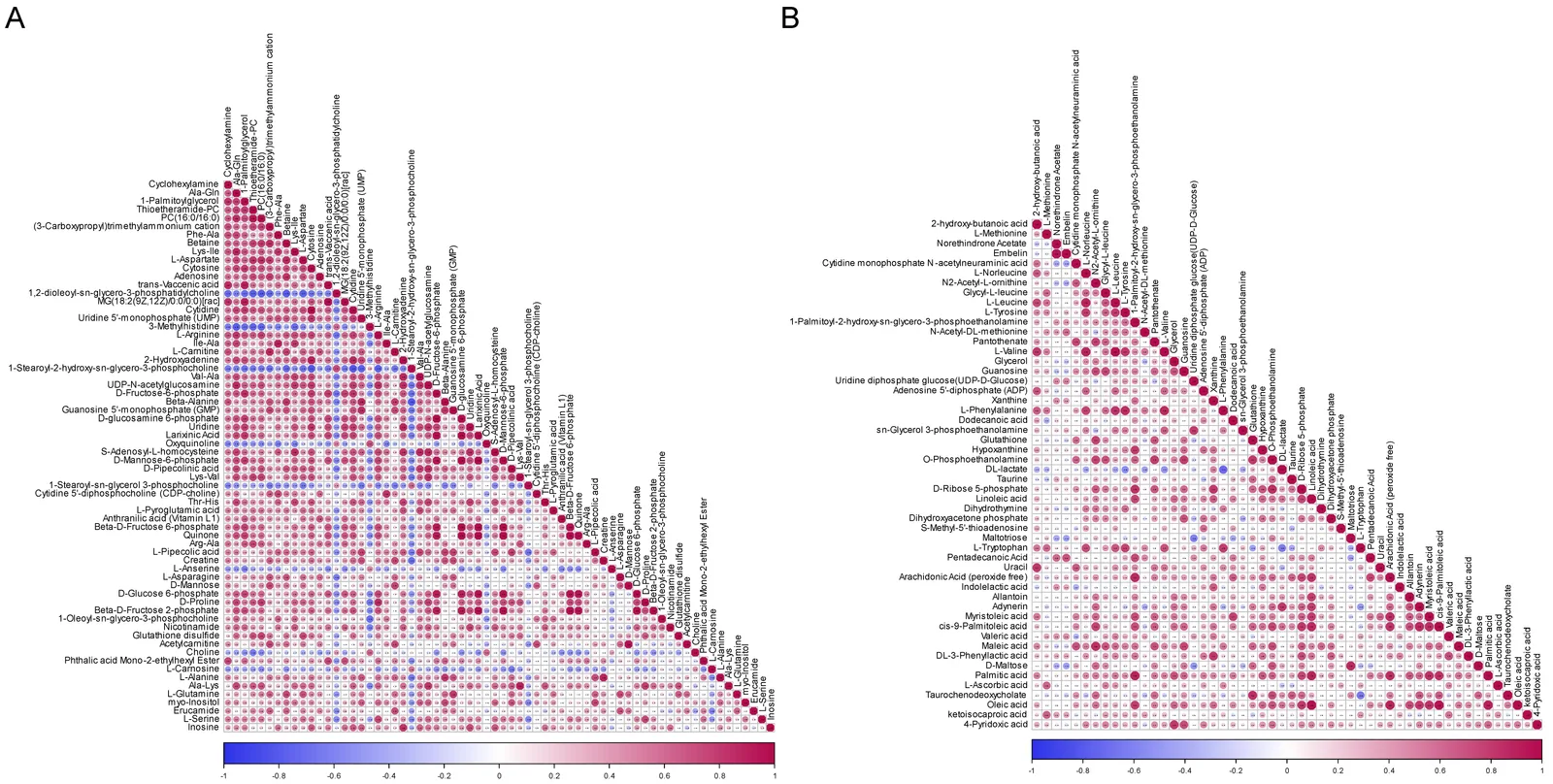
Correlation analysis of DMs. (**A**) Correlation analysis of metabolites in positive ion mode; (**B**) Correlation analysis of metabolites in negative ion mode.

Correlation analysis of the DMs is shown in Figure 3. In positive ion mode, the 9 DMs that were upregulated in Daweishan mini chickens (3-methylhistidine, choline, L-carnosine, L-phenylalanine, 1,2-dioleoyl-sn-glycero-3-phosphatidylcholine, L-anserine, oxyquinoline, 1-stearoyl-2-hydroxy-sn-glycero-3-phosphocholine, and 1-stearoyl-sn-glycerol-3-phosphocholine) were positively correlated with each other, and the 59 downregulated DMs were also positively correlated with each other; however, the 9 upregulated DMs were negatively correlated with the 59 downregulated DMs. In negative ion mode, the 8 DMs that were upregulated in Daweishan mini chickens (2-oxoadipic acid, DL-lactate, L-anserine, L-carnosine, norethindrone acetate, embelin, L-ascorbic acid, and adipic acid) showed similar positive correlations among themselves and negative correlations with the 53 downregulated DMs.

**Figure 4:**
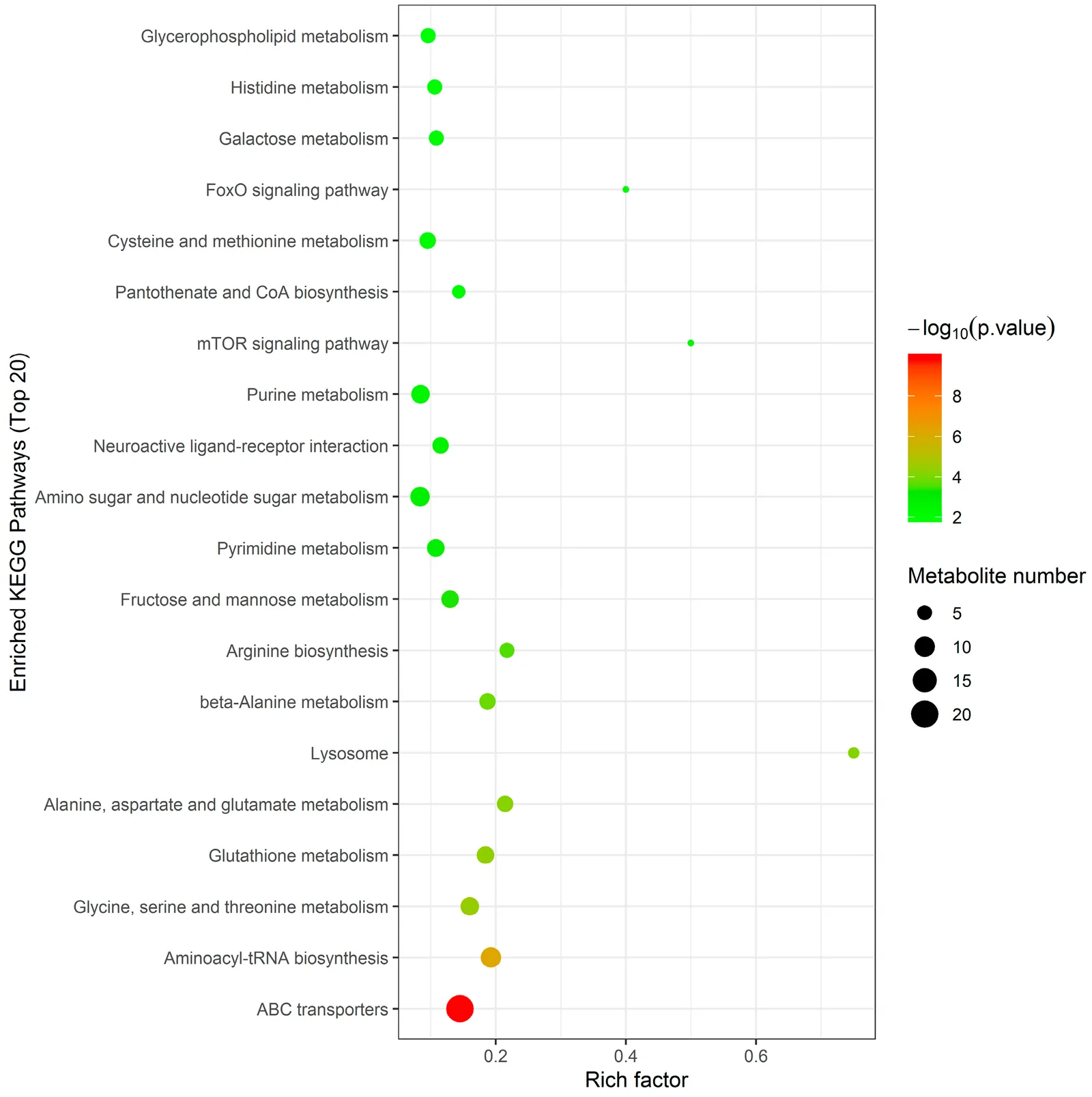
KEGG pathway enrichment analysis of DMs.

KEGG pathway enrichment analysis showed that DMs from the breast muscle tissue of the two chicken breeds were significantly enriched in 26 pathways (*p* < 0.05; Table 2 and Figure 4), including ABC transporters; aminoacyl-tRNA biosynthesis; glycine, serine and threonine metabolism; glutathione metabolism; alanine, aspartate and glutamate metabolism; lysosomes; beta-alanine metabolism; arginine biosynthesis; fructose and mannose metabolism; pyrimidine metabolism; amino sugar and nucleotide sugar metabolism; neuroactive ligand–receptor interaction; purine metabolism; the mTOR signaling pathway; pantothenate and CoA biosynthesis; cysteine and methionine metabolism; the FoxO signaling pathway; galactose metabolism; histidine metabolism; glycerophospholipid metabolism; phenylalanine, tyrosine and tryptophan biosynthesis; the pentose phosphate pathway; glyoxylate and dicarboxylate metabolism; D-arginine and D-ornithine metabolism; D-glutamine and D-glutamate metabolism; and glycolysis/gluconeogenesis.

## 4. Discussion

Poultry meat quality is a comprehensive trait that primarily encompasses sensory quality and nutritional quality (protein, amino acids, and fatty acids). Muscle metabolites are the molecular basis of these qualities: amino acid metabolites such as L-glutamic acid are closely related to the umami taste and tenderness of meat, whereas intramuscular fat and phospholipid contents directly affect juiciness and the formation of flavor precursors. Non-volatile substances such as inosine monophosphate (IMP) and free amino acids determine the intensity of umami perception by activating taste receptors. IMP is the key flavor compound responsible for the umami taste of chicken meat and plays a dominant role in umami formation. Umami amino acids, such as aspartic acid (Asp) and glutamic acid (Glu), can stimulate taste receptors and generate an umami response. 5′-Nucleotides not only contribute to umami themselves but also act synergistically with IMP, significantly enhancing the umami intensity of chicken meat [11]. Compared with Luhua chickens (LH) and AA broilers, the meat of Beijing-You chickens is rich in essential amino acids, arachidonic acid, IMP, and guanosine monophosphate (GMP); furthermore, the ratios of unsaturated fatty acids to saturated fatty acids (2.31) and of polyunsaturated fatty acids to monounsaturated fatty acids (1.52) in Beijing-You chickens are the highest among the three breeds [12].

Amino acids and fats are important components that influence meat quality, which is primarily composed of volatile organic compounds (VOCs) and taste-active substances. VOCs (such as aldehydes, alcohols, and esters) originate from pathways including lipid oxidation, the Maillard reaction, and thiamine thermal degradation [13]. Cooking chicken can produce more than 600 types of VOCs. Among these, aldehydes (e.g., (E,E)-2,4-decadienal), sulfur-containing compounds (e.g., 2-methyl-3-furanthiol), and lipid oxidation products (e.g., hexanal) are core aroma-contributing components, and their concentrations and thresholds jointly determine the overall flavor profile [14,15]. Non-volatile substances act as flavor precursors, regulating the synergistic formation of taste and aroma through thermal reactions; IMP is a key umami substance, and its synergistic enhancement effect with glutamic acid can increase the umami intensity of chicken broth [16]. Numerous studies have demonstrated that the meat quality of indigenous chicken breeds differs significantly from that of modern fast-growing commercial broiler breeds in sensory characteristics such as color, aroma, and taste. The IMP content in the breast muscle of indigenous breeds is significantly higher than that in commercial broilers [5,17]. Differences in the free amino acid profile also influence the flavor characteristics of indigenous breeds; Beijing-You chickens have higher levels of glutamic acid and aspartic acid, whereas commercial broilers are richer in growth-related metabolites such as taurine [18,19]. These flavor precursors generate volatile compounds through the Maillard reaction, lipid oxidation, and their interactions, forming a complex flavor network [16].

In this study, 150-day-old Daweishan mini chickens showed higher abundances of 3-methylhistidine, choline, L-carnosine, L-phenylalanine, L-anserine, DL-lactate, and L-ascorbic acid than in 45-day-old Tegel broilers; these metabolites are closely related to muscle metabolism and meat quality. 3-Methylhistidine, a methylated derivative of myofibrillar proteins (actin/myosin), is liberated during muscle protein degradation and cannot be recycled. It acts as a quantitative marker of myofibrillar protein breakdown and mirrors muscle metabolic turnover [20]. L-Carnosine is a dipeptide (β-alanyl-L-histidine) that acts as a physiological pH buffer in muscle (slowing post-mortem pH decline) and as an antioxidant (scavenging free radicals and inhibiting lipid oxidation), directly improving meat color, water-holding capacity, and flavor stability; it also promotes muscle cell differentiation [21]. L-Anserine is a methylated derivative of carnosine (β-alanyl-3-methyl-L-histidine) found at high concentrations in poultry muscle; its functions are similar to those of carnosine (antioxidant and pH buffering), and it can significantly enhance muscle cell differentiation and contractility through mechanisms involving the histamine H1 receptor (H1R) [22]. DL-Lactate is the end product of post-mortem muscle glycolysis, and its accumulation directly causes the decline in muscle pH, a process that determines meat color, water-holding capacity, and tenderness [23]. Choline is a key component of lecithin (phosphatidylcholine), ensures cell membrane integrity, is a precursor of acetylcholine in nerve signal transmission, and participates in hepatic lipid transport (preventing fatty liver); it influences the lipid composition of muscle membranes and, indirectly, meat quality [24]. L-Phenylalanine is an essential aromatic amino acid and a precursor of tyrosine, dopamine, and norepinephrine; it participates in the Maillard reaction to generate volatile aroma compounds, and its free form influences meat taste [25]. L-Ascorbic acid is a potent antioxidant that protects muscle cells from oxidative damage and is an essential cofactor for collagen synthesis, maintaining connective tissue structure and influencing meat tenderness and connective tissue strength [26]. Other DMs with higher abundances in Daweishan mini chickens included 2-oxoadipic acid, a key intermediate in the catabolism of L-tryptophan, L-lysine, and hydroxylysine that is ultimately converted to glutaryl-CoA for energy metabolism; the phospholipids 1,2-dioleoyl-sn-glycero-3-phosphatidylcholine (DOPC), 1-stearoyl-2-hydroxy-sn-glycero-3-phosphocholine, and 1-stearoyl-sn-glycerol-3-phosphocholine, which are structural components of cell membranes.

The abundances of other DMs, including amino acids and their derivatives, small peptides, carbohydrates and their phosphate esters, nucleotides and their derivatives, lipids and their metabolites, and organic acids, amines, and other nitrogen-containing compounds, were lower in 150-day-old Daweishan mini chickens than in 45-day-old Tegel broilers. During animal development, these components form a precisely coordinated network that drives tissue construction through energy supply, material synthesis, and signal regulation, exerting a significant influence on meat quality. These DMs were enriched in KEGG signaling pathways related to meat quality, including aminoacyl-tRNA biosynthesis; glycine, serine and threonine metabolism; glutathione metabolism; alanine, aspartate and glutamate metabolism; arginine biosynthesis; purine metabolism; pantothenate and CoA biosynthesis; cysteine and methionine metabolism; glycerophospholipid metabolism; and D-glutamine and D-glutamate metabolism. Aminoacyl-tRNA biosynthesis is the initial step of protein translation, ensuring the accurate transport of amino acids to ribosomes for protein synthesis; the activity of this pathway directly determines the efficiency of muscle protein deposition, thereby affecting meat tenderness and water-holding capacity [27]. Glycine, serine, and threonine metabolism is one of the key pathways for the formation of meat flavor precursors. These amino acids not only have taste-active properties themselves (glycine has a sweet taste) but also participate in the synthesis of important biomolecules such as glutathione, purines, and heme. As a component of glutathione, glycine indirectly contributes to meat flavor formation; in addition, the catabolism of threonine generates glycine and acetyl-CoA, connecting carbohydrate metabolism with amino acid metabolism. The activity of this pathway is closely related to the composition and content of free amino acids in meat and is an important factor determining the final flavor richness of meat products [28]. Glutathione is an important flavor precursor substance; its antioxidant effect helps delay lipid oxidation in post-slaughter muscle, maintain meat color stability, and extend shelf life. Glutathione metabolism directly affects the redox balance in muscle, correlating with meat tenderness, juiciness, and flavor quality; the normal functioning of this pathway is of great significance for maintaining meat freshness and flavor formation [29]. Alanine, aspartate, and glutamate metabolism is directly related to the formation of umami in meat: glutamate is a well-known umami substance, aspartate also enhances umami, and alanine contributes a sweet taste; together, they determine the basic taste of meat [30]. This pathway also connects carbohydrate metabolism and amino acid metabolism by introducing carbon skeletons into the tricarboxylic acid cycle through transamination, providing support for energy metabolism. Therefore, alanine, aspartate, and glutamate metabolism is a core pathway regulating the taste quality and freshness of meat products. Arginine biosynthesis achieves endogenous arginine supply through the citrulline–arginine shuttle and plays an important regulatory role in meat quality; activation of this pathway promotes the accumulation of umami compounds (L-glutamate and L-glutamine), thereby enhancing the umami taste of meat [31]. Purine metabolism is one of the key pathways determining the umami taste and nutritional value of meat: its metabolite IMP is the primary umami substance, while GMP also has an umami-enhancing effect, and the dynamic changes in purine metabolism directly determine the final flavor quality of meat products, especially the balance between umami and bitterness [32]. Pantothenate (vitamin B5) is a precursor for the synthesis of coenzyme A (CoA), an indispensable cofactor in the metabolism of carbohydrates, fatty acids, and amino acids; pantothenate deficiency can lead to growth retardation and abnormal fat metabolism, affecting animal production performance and meat quality. CoA participates in the tricarboxylic acid cycle and fatty acid β-oxidation, providing energy for muscle activity and protein synthesis, and is an essential factor for the synthesis of cholesterol and triglycerides, indirectly influencing intramuscular fat deposition [33]. Cysteine and methionine are sulfur-containing amino acids whose metabolism is closely related to meat flavor and antioxidant capacity; by providing flavor precursors and antioxidant protection, they comprehensively affect the flavor and quality stability of meat products [34]. Glycerophospholipid metabolism is directly related to lipid oxidation and the generation of flavor precursors: glycerophospholipids are the main components of biological membranes, and their degradation during heating is a major source of volatile aroma compounds (such as aldehydes and ketones), contributing significantly to the overall aroma of meat products, especially the fatty aroma [35]. D-glutamine and D-glutamate metabolism involves the conversion of D-form glutamate and glutamine. L-Glutamate is a key umami substance, and L-glutamine participates in nitrogen transport and antioxidant defense; glutamine can be converted to glutamate via glutaminase, increasing the reserve of umami precursors [30].

As a key time dimension for the growth and development of chickens, age directly regulates the metabolic network of muscle tissue, which in turn affects the quality and nutritional characteristics of chicken. During the growth cycle from chick to adult chicken, the composition and content of muscle metabolites change in stages, and the core differences are reflected in the dynamic adjustment of energy substances, amino acids, and flavor precursors. In the chick stage, muscle metabolism is centered on rapid energy supply [36], and amino acid metabolism is mainly based on the rapid synthesis and utilization of essential amino acids, which are used to build the basic protein framework of muscle tissue; consequently, the total amount of free amino acids in muscle is low. In the fattening stage, the focus of muscle metabolism shifts to fat deposition and protein optimization: lipid metabolites such as triglycerides and phospholipids gradually accumulate between muscle fibers, especially unsaturated fatty acids (such as oleic acid and linoleic acid), which directly affect the tenderness and taste of chicken [34]; meanwhile, creatine, inosinic acid, and other nitrogenous metabolites in muscle begin to accumulate. Among them, inosinic acid, as a key flavor precursor, increases significantly with age, laying the foundation for the fresh flavor of chicken. After reaching the slaughter age, muscle metabolism stabilizes and the metabolite composition enters a plateau [37,38].

In this study, the abundances of many DMs in 150-day-old Daweishan mini chickens were lower than those in 45-day-old Tegel broilers. But it should be noted that Daweishan mini chickens and Tegel broilers were sampled at their respective market ages of 150 d and 45 d in the present study. Therefore, slaughter age represents a confounding variable for breed comparison. We cannot disentangle the contributions of the breed effect and the slaughter-age effect, and the observed metabolic differences should not be solely attributed to breed or age. And we have clearly added a dedicated limitation paragraph to acknowledge that metabolite changes observed here cannot directly equate to actual flavor or meat quality phenotypes. Future experiments with uniform slaughter age and meat quality are needed to further dissect breed-specific metabolic characteristics.

## 5. Conclusions

DMs enriched in aminoacyl-tRNA biosynthesis; glycine, serine and threonine metabolism; glutathione metabolism; alanine, aspartate and glutamate metabolism; arginine biosynthesis, purine metabolism; pantothenate and CoA biosynthesis; cysteine and methionine metabolism; glycerophospholipid metabolism; and D-glutamine and D-glutamate metabolism may have contributed to the differences in the quality of 150-day-old Daweishan mini chickens and 45-day-old Tegel broilers. Moreover, 3-methylhistidine, L-carnosine, L-anserine, DL-lactate, choline, L-phenylalanine, and L-ascorbic acid were present at higher relative abundances in the muscles of Daweishan mini chickens at marketable age.

## Figures and Tables

**Table 1:** The result of the permutation test.

	R^2^X (Cum)	R^2^Y (Cum)	Q^2^ (Cum)
Positive	0.301	0.995	0.879
Negative	0.316	0.99	0.861

**Table 2:** Enrichment results of the KEGG pathway analysis of DMs.

Pathway Name	Compound Name	*p*-Value	FDR
ABC transporters	L-Alanine, Glycine, Guanosine, L-Arginine, Uridine, Inosine, L-Glutamine, L-Glutamate, myo-Inositol, D-Maltose, Glutathione, L-Leucine, Betaine, L-Aspartate, Adenosine, Cytidine, D-Mannose, Choline, L-Phenylalanine, L-Serine	1.3 × 10^−10^	8.85 × 10^−9^
Aminoacyl-tRNA biosynthesis	L-Alanine, Glycine, L-Asparagine, L-Arginine, L-Glutamine, L-Glutamate, L-Leucine, L-Aspartate, L-Phenylalanine, L-Serine	5.89 × 10^−7^	2 × 10^−5^
Glycine, serine and threonine metabolism	Glycine, Creatine, Sarcosine, Dimethylglycine, Betaine, L-Aspartate, Choline, L-Serine	3.57 × 10^−5^	0.00075
Glutathione metabolism	5-L-Glutamyl-L-alanine, Glycine, L-Ascorbic acid, L-Glutamate, L-Pyroglutamic acid, Glutathione disulfide, Glutathione	4.41 × 10^−5^	0.00075
Alanine, aspartate and glutamate metabolism	L-Alanine, L-Asparagine, L-Glutamine, L-Glutamate, L-Aspartate, D-glucosamine 6-phosphate	6.55 × 10^−5^	0.000782
Lysosome	Adenosine 5′-diphosphate (ADP), D-Mannose-6-phosphate, D-Mannose	6.9 × 10^−5^	0.000782
beta-Alanine metabolism	Pantothenate, Uracil, L-Anserine, L-Carnosine, L-Aspartate, Beta-Alanine	0.000145	0.001407
Arginine biosynthesis	N2-Acetyl-L-ornithine, L-Arginine, L-Glutamine, L-Glutamate, L-Aspartate	0.000259	0.0022
Fructose and mannose metabolism	Beta-D-Fructose 6-phosphate, Dihydroxyacetone phosphate, Alpha-D-Glucose, D-Mannose 1-phosphate, D-Mannose-6-phosphate, D-Mannose, Beta-D-Fructose 2-phosphate	0.00044	0.003327
Pyrimidine metabolism	Uridine 5′-monophosphate (UMP), Uridine, Uracil, L-Glutamine, Cytosine, Cytidine, Beta-Alanine	0.001371	0.00932
Amino sugar and nucleotide sugar metabolism	N-Acetyl-D-Glucosamine 6-Phosphate, Cytidine monophosphate N-acetylneuraminic acid, UDP-N-acetylglucosamine, Beta-D-Fructose 6-phosphate, Alpha-D-Glucose, D-Mannose 1-phosphate, D-glucosamine 6-phosphate, D-Mannose-6-phosphate, D-Mannose	0.001823	0.011272
Neuroactive ligand-receptor interaction	Glycine, L-Glutamate, Adenosine 5′-diphosphate (ADP), L-Aspartate, Adenosine, Beta-Alanine	0.002137	0.012112
Purine metabolism	Guanosine 5′-monophosphate (GMP), Glycine, Guanosine, Inosine, L-Glutamine, D-Ribose 5-phosphate, Adenosine 5′-diphosphate (ADP), Adenosine	0.003089	0.016158
mTOR signaling pathway	L-Arginine, L-Leucine	0.003966	0.019266
Pantothenate and CoA biosynthesis	Pantothenate, Uracil, L-Aspartate, Beta-Alanine	0.005638	0.024085
Cysteine and methionine metabolism	L-Alanine, S-Adenosyl-L-homocysteine, Glutathione, L-Aspartate, S-Methyl-5′-thioadenosine, L-Serine	0.005667	0.024085
FoxO signaling pathway	L-Glutamate, Adenosine 5′-diphosphate (ADP)	0.006498	0.024797
Galactose metabolism	Dihydroxyacetone phosphate, Alpha-D-Glucose, myo-Inositol, D-Fructose-6-phosphate, D-Mannose	0.006564	0.024797
Histidine metabolism	L-Glutamate, L-Anserine, L-Carnosine, L-Aspartate, 3-Methylhistidine	0.007196	0.025755
Glycerophospholipid metabolism	Dihydroxyacetone phosphate, O-Phosphoethanolamine, Cytidine 5′-diphosphocholine (CDP-choline), PC(16:0/16:0), Choline	0.011007	0.037422
Phenylalanine, tyrosine and tryptophan biosynthesis	D-Fructose 1,6-bisphosphate, D-Erythrose 4-phosphate, Anthranilic acid (Vitamin L1), L-Phenylalanine	0.012542	0.038767
Pentose phosphate pathway	D-Erythrose 4-phosphate, Beta-D-Fructose 6-phosphate, 2′-Deoxy-D-ribose, D-Ribose 5-phosphate	0.012542	0.038767
Glyoxylate and dicarboxylate metabolism	L-Malic acid, Glycine, L-Glutamine, L-Glutamate, L-Serine	0.02235	0.066077
D-arginine and D-ornithine metabolism	D-Ornithine, L-Arginine	0.032257	0.091393
D-glutamine and D-glutamate metabolism	L-Glutamine, L-Glutamate	0.044221	0.120281
Glycolysis/Gluconeogenesis	Beta-D-Fructose 6-phosphate, Dihydroxyacetone phosphate, Alpha-D-Glucose	0.046512	0.121648

## Data Availability

The data presented in this study are available on request from the corresponding author. The data are not publicly available due to institutional ethical restrictions on sharing animal experiment data.

## Supplementary Materials

The following supporting information can be downloaded at: https://www.mdpi.com/article/10.3390/biology15201804/s1. Table S1: DMs in positive ion mode; Table S2: DMs in negative ion mode.
